# Genomic characteristics of two most widely used BCG vaccine strains: Danish 1331 and Pasteur 1173P2

**DOI:** 10.1186/s12864-022-08826-9

**Published:** 2022-08-21

**Authors:** Mahla Asadian, Seyed Mehdi Hassanzadeh, Azadeh Safarchi, Masoumeh Douraghi

**Affiliations:** 1 Division of Microbiology, Department of Pathobiology, School of Public Health, Tehran University of Medical Sciences, Tehran, Iran; 2 BCG Vaccine Production Plant, Pasteur Institute of Iran, Karaj, Iran; 3 School of Biotechnology and Biomolecular Sciences, University of New South Wales, Sydney, Australia

**Keywords:** BCG vaccine, Danish 1331, Genomic analysis, Pasteur 1173P2

## Abstract

**Background:**

Bacillus Calmette–Guérin (BCG) refers to a group of vaccine strains with unique genetic characteristics. BCG is the only available vaccine for preventing tuberculosis (TB). Genetic and biochemical variations among the BCG vaccine strains have been considered as one of the significant parameters affecting the variable protective efficacy of the vaccine against pulmonary tuberculosis. To track genetic variations, here two vaccine strains (Danish 1331 and Pasteur 1173P2) popularly used according to the BCG World Atlas were subjected to a comparative analysis against the *Mycobacterium tuberculosis* H37Rv, *Mycobacterium bovis* AF2122/97, and *Mycobacterium tuberculosis* variant bovis BCG str. Pasteur 1173P2 reference genomes. Besides, the presence or absence of the experimentally verified human T cell epitopes was examined.

**Results:**

Only two variants were identified in BCG Danish 1331 that have not been reported previously in any BCG strains with the complete submitted genome yet. Furthermore, we identified a DU1-like 14,577 bp region in BCG Danish 1331; The duplication which was previously seemed to be exclusive to the BCG Pasteur. We also found that 35% of the T cell epitopes are absent from both strains, and epitope sequences are more conserved than the rest of the genome.

**Conclusions:**

We provided a comprehensive catalog of single nucleotide polymorphisms (SNPs) and short insertions and deletions (indels) in BCG Danish 1331 and BCG Pasteur 1173P2. These findings may help determine the effect of genetic variations on the variable protective efficacy of BCG vaccine strains.

**Supplementary Information:**

The online version contains supplementary material available at 10.1186/s12864-022-08826-9.

## Background

Bacillus Calmette-Guérin (BCG), an attenuated derivative of *Mycobacterium bovis* (*M. bovis*), is the only vaccine used against tuberculosis. It is obtained through 230 consecutive in vitro passages over 13 years at the Pasteur Institute of Lille in 1921 [1]. The vaccine has been used to immunize more than four billion people over a century, which has made BCG the most widely used vaccine [1]. In 1924, the primary BCG vaccine was distributed to different countries, and the continuous subcultures under different conditions led to the emergence of various vaccine strains. Until the development of the seed lot system in the 1960s, BCG vaccine strains were exposed to more than 1000 passages in different laboratories that resulted in genotypic and phenotypic differences among them [2, 3].

Although the role of BCG has been proved in the prevention of disseminated forms of tuberculosis in children [4, 5], its protective efficacy is highly variable against pulmonary tuberculosis in adults (0%-80%) [6, 7]. Several factors, such as the usage of different vaccine strains with genetic differences, contact with non-tuberculosis mycobacteria (NTMs), host genetic factors, and diversities in the circulating *Mycobacterium tuberculosis* (*M. tuberculosis*) strains are associated with variability in protection [8–11].

While there is evidence about the evolution of the BCG vaccine strains since 1921 [1, 2], the genetic differences among these strains as one of the most important factors that might affect immunogenicity, viability, virulence, and thus variable efficacy have been little investigated. The genetic events may be shared among all strains and appear to be involved in attenuation of the primary strain or may be observed in each of the vaccine strains exclusively and be responsible for their over-attenuation [12–18]. Therefore, there is a need to examine the frequency of these genetic differences and their relationship with the phenotype of vaccines used by comparing the whole genome sequences of the BCG vaccine strains.

In 2009 and 2010, to standardize the vaccine production, Danish 1331, Tokyo 172–1, Russia BCG-1, and Moreau-RJ strains were introduced by the WHO Expert Committee on Biological Standardization (ECBS) as BCG reference strains [19, 20]. However, the chosen vaccine strain is different in the various countries and there is insufficient evidence to prove which strain provides the best protective efficacy against tuberculosis [21]. According to the latest update of the BCG World Atlas in 2020, Danish 1331 (16.6%), Pasteur 1173P2 (9.2%) and, Tokyo 172 (7.3%) strains are the most globally used strains for vaccine production, respectively [21]. This study aimed to analysis the complete genome sequences of the two most widely used BCG vaccine strains, including the reference strain introduced by WHO (Danish 1331) and the BCG strain used in Iran (Pasteur 1173P2). The main hypothesis of this study was to identify the new genetic variations in these two BCG strains that have not been reported so far in comparison to the reference strains.

## Results

### Genomic features for BCG Pasteur 1173P2 and BCG Danish 1331

**Fig. 1:**
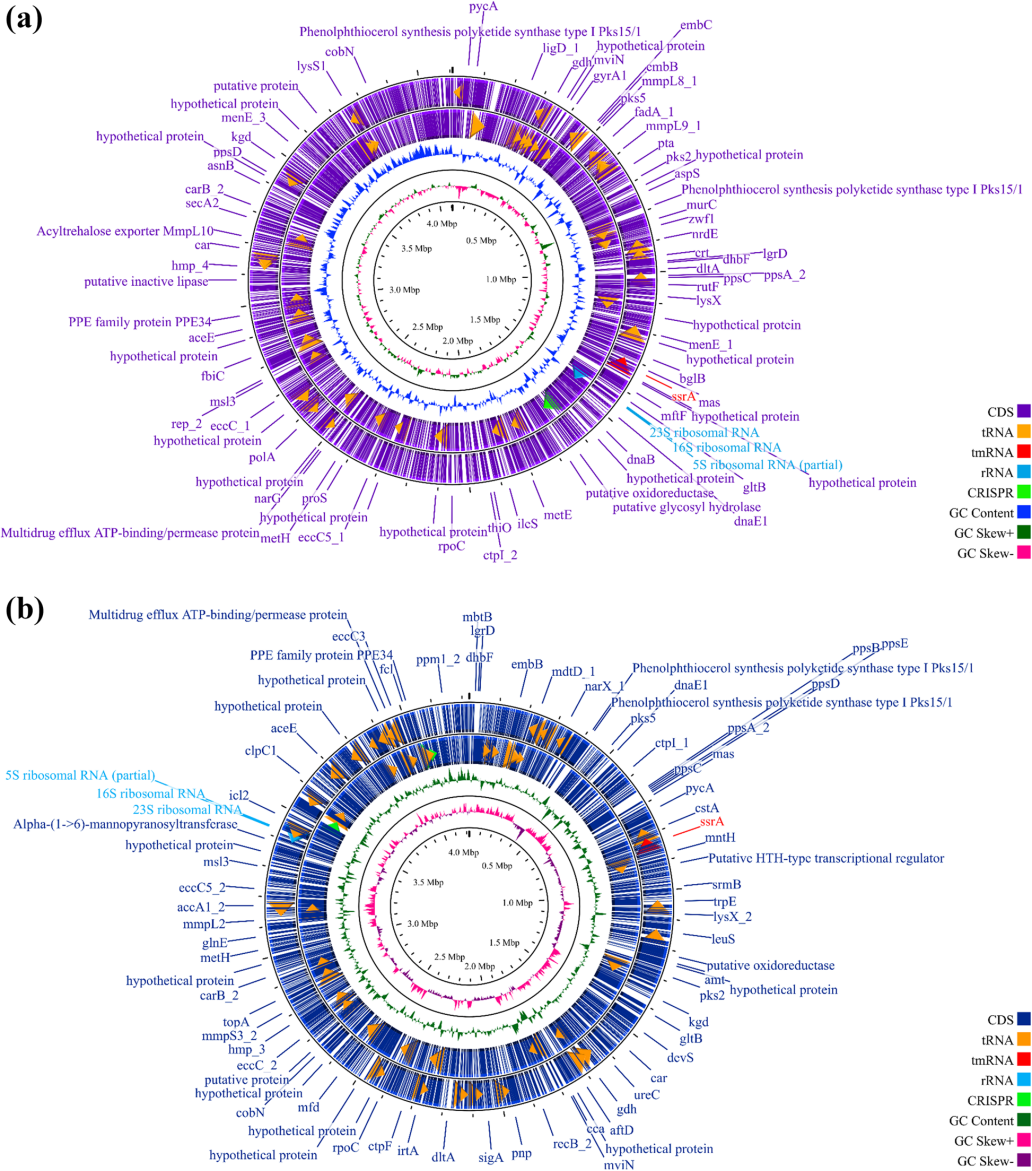
General genomic features of the BCG Pasteur 1173P2 and BCG Danish 1331. **a** Circular representation of the BCG Pasteur 1173P2 contigs using Proksee (https://proksee.ca). The scale is shown in megabases on the black central circle. Moving inward, two outer violet circles show forward and reverse strand CDSs, respectively. Some genes are shown on the outer violet circle with the Proksee's default. The tRNAs (orange arrows), rRNAs (light blue arrows), tmRNA (red arrow) and two CRISPR sequences (light green arrows adjacent each other) are shown in CDSs circles. The next circle shows GC content (dark blue) followed by the GC skew (dark green and pink). **b** Circular representation of the BCG Danish 1331 contigs using Proksee (https://proksee.ca). The scale is shown in megabases on the black central circle. Moving inward, two outer dark blue circles show forward and reverse strand CDSs, respectively. Some genes are shown on the outer dark blue circle with the Proksee's default. The tRNAs (orange arrows), rRNAs (light blue arrows), tmRNA (red arrow) and two CRISPR sequences (light green arrows) are shown in CDSs circles. The next circle shows GC content (dark green) followed by the GC skew (violet and pink). Category 0 > Virulence, detoxification, adaptation. Category 1 > Lipid metabolism. Category 2 > Information pathways. Category 3 > Cell wall and cell processes. Category 5 > Insertion sequences and phages. Category 6 > PE/PPE. Category 7 > Intermediary metabolism and respiration. Category 8 > Unknown. Category 9 > Regulatory proteins. Category 10 > Conserved hypothetical proteins. Category 16 > Conserved hypothetical with an orthologous in *M. bovis*

The quality control of the assemblies estimated the total length of the contigs to be 4.2 Mbp for Pasteur 1173P2 and 4.3 Mbp for Danish 1331, and the GC content approximately 65% for both strains. A total of 4060 genes for Pasteur 1173P2 and 4037 for Danish 1331 were identified through scaffolds annotation (Fig. 1a-b). Total coding sequences (CDSs) and tRNA genes were found 4006 and 50 for Pasteur 1173P2 and 3982 and 51 for Danish 1331, respectively. Three genes for rRNA (5S, 16S, and 23S) and one for tmRNA (*ssr*A) were identified in both strains (Fig. 1a-b). In comparison to *M. tuberculosis* H37Rv, 58 and 31 genes encoding PPE and PE family proteins were recognized in both strains, respectively. In addition, 58 genes encoding proteins of the PE_PGRS subfamily were identified in Pasteur 1173P2 and 59 in Danish 1331. Among the regions of difference (RDs) that can differentiate between these two late strains, RD14 and N-RD18 were identified in Pasteur 1173P2 while not exist in Danish 1331. Moreover, we identified a DU1-like 14,577 bp region in Danish 1331.

### Genetic variations in comparison to *M. tuberculosis* H37Rv

The mean nucleotide variations (SNPs and Indels below 100 bp) was 549.7 for Pasteur 1173P2 and 559.9 for Danish 1331 per megabase of contigs length. In comparing the genome sequence of *M. tuberculosis* H37Rv with those of Pasteur 1173P2 and Danish 1331, a total of 2289 SNPs was identified (Table S1). The 2155 SNPs were shared in both strains, while 56 were found in Pasteur 1173P2 and 78 in Danish 1331. From 1992 SNPs occurred within 1328 genes, 63.8% are non-synonymous. Non-synonymous SNPs (nSNP) were found in 28 genes that resulted in translation shift compared to their homolog in *M. tuberculosis* H37Rv. Substitution of X404Q in surface-associated esterase LipC leads to a longer protein and nonsense mutations in *ugp*B (Rv2833c) and *lpd*A (Rv3303c) result in the lack of functional proteins. Analysis of strain-specific SNPs revealed only a nonsense mutation in *gal*E2 (Rv0501) and an nSNP in Rv1830 in the Danish 1331.

Consideration of genes encoding regulatory proteins in both strains revealed a mutation in *vir*S (Rv3082c) that could affect the transcription of the *mym*A operon (Rv3089-Rv3083). Mutations were also found in *crp* (Rv3676), *pst*P (Rv0018c), and *fha*A -as a mycobacterial core gene- (Rv0020c). Members of the mycobacterial serine/threonine protein kinases family (PknH, PknF, PknL, and PknK) and two-component systems (SenX3-RegX3, MprAB, KdpDE, etc.) were also identified in the regulators containing variants. *pks*12, as the largest open reading frame (ORF) in the mycobacterial genome, showed the most nSNPs among genes. In total, 47 nSNPs were detected in genes specifically required for mycobacterial in vivo survival in both strains (Table 1). Non-synonymous mutations were also identified in sigma factors involved in the initiation of replication. We found the substitution of guanine residue by adenine in the initiation codon of *sig*K (Rv0445c) and a nonsense mutation in Rv3687c encoding the anti-sigma factor antagonist RsfB. SNPs were also found in loci encoding ribosomal proteins in both strains. These mutations, which cause allelic differences between *M. tuberculosis* and BCG, lead to an altered amino acid at only four loci.

**Table 1:** Required genes for mycobacterial in vivo growth that contain non-synonymous SNPs in Pasteur 1173P2 and Danish 1331

No	H37Rv locus tag	Gene name	Product	Non-synonymous amino acid substitution	Pasteur 1173P2	Danish 1331
1	Rv0101	*nrp*	Peptide synthetase Nrp	L1365M	+	+
2	Rv0169	*mce*1A	Mce family protein Mce1A	S313A, P359S	+	+
3	Rv0170	*mce*1B	Mce family protein Mce1B	I179T	+	+
4	Rv0171	*mce*1C	Mce family protein Mce1C	E212D	+	+
5	Rv0176	-	Mce associated transmembrane protein	N285S, S291A	+	+
6	Rv0218	-	Transmembrane protein	C316R, D413N	+	+
7	Rv0490	*sen*X3	Two component sensor histidine kinase SenX3	F109S	+	+
8	Rv0636	*had*B	(3R)-hydroxyacyl-ACP dehydratase subunit HadB	T54A	+	+
9	Rv0643c	*mma*A3	Methoxy mycolic acid synthase MmaA3	G98D	+	+
10	Rv1028c	*kdp*D	Sensor protein KdpD	N776D, P368S, G295D, P83S	+	+
11	Rv1109c	-	Hypothetical protein	A147T	+	+
12	Rv1128c	-	Hypothetical protein	E270G	+	+
13	Rv1204c	-	Hypothetical protein	L484I	+	+
14	Rv1224	*tat*B	Sec-independent protein translocase protein TatB	W8G	+	+
15	Rv1244	*lpq*Z	Lipoprotein LpqZ	Q242K	+	+
16	Rv1338	*mur*I	Glutamate racemase	R154L	+	+
17	Rv1371	-	Membrane protein	I368V	+	+
18	Rv1460	-	Transcriptional regulator	I198F, A266V	+	+
19	Rv1640c	*lys*X	Bifunctional lysine-tRNA ligase/phosphatidylglycerol lysyltransferase	D944G, D769E	+	+
20	Rv2048c	*pks*12	Polyketide synthase	A4047S, P3095L, S2964R H2147Q, G1865S	+	+
21	Rv2072c	*cob*L	Precorrin-6Y C(5,15)-methyltransferase	L205P	+	+
22	Rv2275	-	Cyclo(L-tyrosyl-L-tyrosyl) synthase	E261A	+	+
23	Rv2359	*zur*	Zinc uptake regulation protein	H64R	+	+
24	Rv2374c	*hrc*A	Heat-inducible transcription repressor HrcA	R79Q	+	-
25	Rv2388c	*hem*N	Oxygen-independent coproporphyrinogen III oxidase	A184T	+	+
26	Rv2483c	*pls*C	Bifunctional L-3 phosphoserine phosphatase/1-acyl-sn-glycerol-3-phosphate acyltransferase	C189G	+	+
27	Rv2502c	*acc*D1	Acetyl/propionyl-CoA carboxylase subunit beta	F343L, G77S	+	+
27	Rv2692	*ceo*C	TRK system potassium uptake protein CeoC	I133V	+	+
29	Rv2696c	-	Hypothetical protein	D164N	+	+
30	Rv2702	*ppg*K	Polyphosphate glucokinase	I203T	+	+
31	Rv2813	*-*	Hypothetical protein	I76V	+	+
32	Rv2845c	*pro*S	Proline–tRNA ligase	A232T, H177R	+	+
33	Rv2936	*drr*A	Daunorubicin ABC transporter ATP-binding protein DrrA	H309D	+	+
34	Rv2981c	*ddl*A	D-alanine–D-alanine ligase	T365A	+	+
35	Rv3042c	*ser*B2	Phosphoserine phosphatase SerB	G116E, A70S	+	+
36	Rv3061c	*fad*E22	Acyl-CoA dehydrogenase FadE22	S497C, K488E	+	+
37	Rv3087	-	Diacylglycerol O-acyltransferase	L447V	+	+
38	Rv3114	-	Hypothetical protein	S11P	+	+
39	Rv3277	-	Transmembrane protein	S272L	+	+
40	Rv3335c	-	Integral membrane protein	A86V	+	+
41	Rv3371	-	Diacylglycerol O-acyltransferase	R339G, I368V	+	+
42	Rv3497c	*mce*4C	Mce family protein Mce4C	T46P	+	+
43	Rv3551	-	CoA-transferase subunit alpha	A7S	+	+
44	Rv3563	*fad*E32	Acyl-CoA dehydrogenase FadE32	Q105R, W275S	+	+
45	Rv3616c	*esp*A	ESX-1 secretion-associated protein EspA	T192I, A4V	+	+
46	Rv3805c	*aft*B	Terminalbeta-(1- > 2)-arabinofuranosyltransferase	I327V	+	+
47	Rv3868	*ecc*A1	ESX-1 secretion system protein EccA1	A243V	+	+
48	Rv3910	-	Peptidoglycan biosynthesis protein	V480A	+	+

A total of 222 indels below 100 bp were detected, of which 199 occurred in both strains, 14 in Danish 1331 and nine in Pasteur 1173P2 (Table S2). In total, deletions accounted for 53.1% and insertions 46.9%. Of the 154 indels in the genes, 115 led to frameshift mutations and abnormally short and long proteins. Danish 1331 was found as a *pho*R mutant due to a 10 bp deletion, whereas this loss was not detected for Pasteur 1173P2. Indels identified in both strains included frameshift insertions in *mce*1R (Rv0165c) and *pkn*D (Rv0931c) encoding transcriptional regulators belonging to the GntR family and serine/threonine protein kinases. As well, the *pho*T (Rv0820) and *pst*B (Rv0933), which encode members of the ABC transporter complex PstSCAB, *nrd*Z (Rv0570), *rec*B (Rv0630c), *tre*Z (Rv1562c), and *stp* (Rv2333c) have shifted frames. Both strains were identified as *sig*M mutants. Furthermore, in the study of MIRU-VNTR loci, we found that locus 580 located in the intergenic region of the genes encoding the components of the SenX3-RegX3 two-component regulatory system had two and three 77 bp repeats in Pasteur 1173P2 and Danish 1331, respectively.

**Fig. 2:**
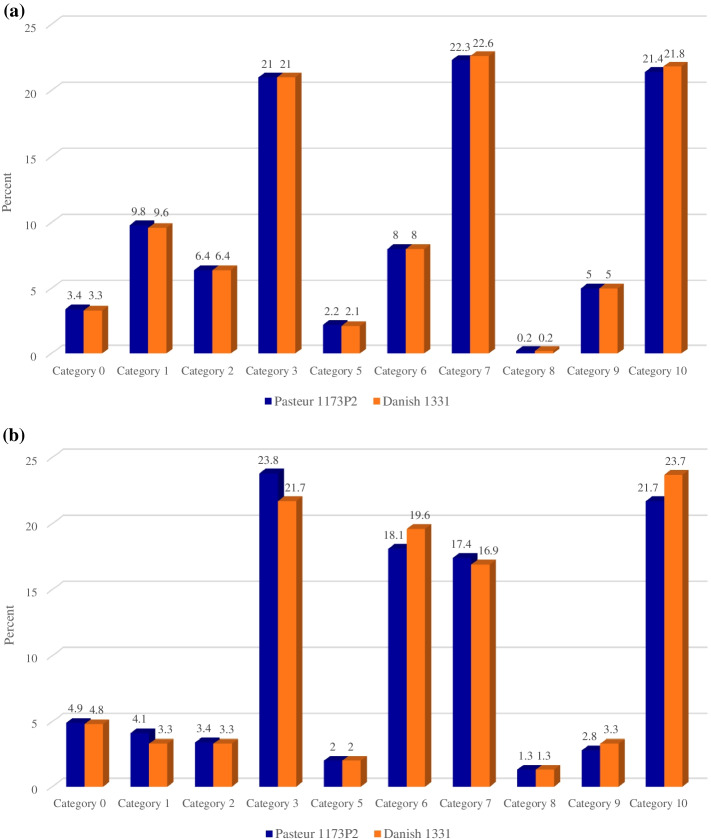
**a** SNPs rate in the functional classification of genes encoding a protein in *M. tuberculosis*. **b** Indels rate in the functional classification of genes encoding a protein in *M. tuberculosis*. Category 0 > Virulence, detoxification, adaptation. Category 1 > Lipid metabolism. Category 2 > Information pathways. Category 3 > Cell wall and cell processes. Category 5 > Insertion sequences and phages. Category 6 > PE/PPE. Category 7 > Intermediary metabolism and respiration. Category 9 > Regulatory proteins. Category 10 > Conserved hypothetical proteins. Category 16 > Conserved hypothetical with an orthologous in *M. tuberculosis*

Assessment of the distribution of the variants in the functional classification of genes encoding a protein in *M. tuberculosis* using TubercuList (http://genolist.pasteur.fr/TubercuList/) showed that SNPs rate in genes involved in intermediate metabolism and respiration is higher in both strains (Fig. 2a). Most indels were detected in Pasteur 1173P2 and Danish 1331 in genes encoding proteins involved in cellular processes and conserved hypotheticals, respectively (Fig. 2b).

### Genetic variations in comparison to *M. bovis* AF2122/97

In comparison to the *M. bovis* AF2122/97 genomic sequence, a set of 728 high-quality SNPs was identified (Table S3). Of these, 645 SNPs occurred in genes and 83 SNPs located in intergenic regions. 672 SNPs were found in both strains, 34 SNPs in Danish 1331 and 22 in Pasteur 1173P2 alone. The 65.6% of SNPs in genes were associated with non-synonymous amino acid substitutions and 34.4% with synonymous. Ten nSNPs cause a change in protein length; Of them, seven SNPs result in the longer protein and three lead to the shorter ones. Several SNPs, including mutation in *gal*E2 (Mb0513) in Danish 1331, were also identified compared to the *M. tuberculosis* H37Rv. In addition, mutations in *lpr*L (Mb0609) and *fad*B3A (Mb1742) lead to longer products than their homolog in *M. bovis*. The missense mutations in *mma*A3 (Mb0662c) and *pyk*A (Mb1643) were also identified in both strains.

Out of 74 indels found, 62 in both strains, five in the Pasteur 1173P2, and seven in the Danish 1331 were identified (Table S4). The 55.4% of indels were insertions and 44.6% deletions. Fifty-seven indels occurred in genes, 16 in intergenic regions, and one insertion in a pseudogene. Among the indels in the genes, 42 were frameshift mutations with one to ten base pair insertions or deletions. Pasteur 1173P2-specific indels including 10 and 15 bp deletions and a single base pair insertion in the genes, and two frameshift insertions in the intergenic regions, which were identified when compared to the *M. tuberculosis* H37Rv. Moreover, the Danish 1331-specific indels were three insertions including one and 5 bp with three deletions including one and 10 bp in the genes and a 3 bp insertion in the intergenic region. Some of these were common with the identified indels compared to the *M. tuberculosis* H37Rv. Analyzes also showed frameshifts in *fus*A2a (Mb0125C), *ugp*B (MB2857C), *ugp*Aa (Mb2860C), and *glp*K (Mb3722C) (Involved in glycerol catabolism) compared to *M. bovis* AF2122/97.

**Fig. 3:**
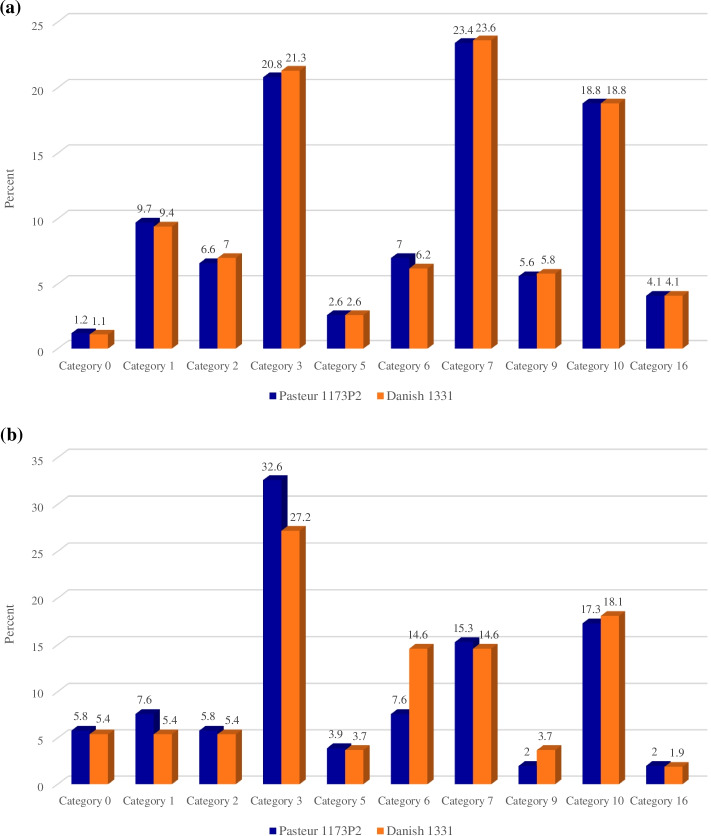
**a** SNPs rate in the functional classification of genes encoding a protein *M. bovis*. **b** Indels rate in the functional classification of genes encoding a protein in *M. bovis*

Using BoviList (http://genolist.pasteur.fr/BoviList/), it was shown that the rate of SNPs and indels in the genes involved in the intermediate metabolism and respiration, and the genes encoding proteins involved in the cell wall and cell processes is higher in both strains, respectively (Fig. 3a-b).

### Genetic variations in comparison to *M. tuberculosis* variant bovis BCG str. Pasteur 1173P2

A total of 37 variants (30 SNPs and 7 Indels) were identified (Table S5). The identified SNPs were three (a synonymous SNP [sSNP] and two SNPs in the intergenic region) for the Pasteur 1173P2 and 30 (14 nSNPs and seven sSNPs in the genes, and nine in the intergenic regions) for Danish 1331. Most of the SNPs identified in Danish 1331 compared to Pasteur 1173P2 were also found as Pasteur-SNPs compared to *M. bovis* AF2122/97. A missense mutation was observed in RS02785, encoding the oxidoreductase of the SDR family in Danish 1331. An insertion was shared between two strains, which was in frame. Three indels in the genes showed frameshift mutations in Danish 1331. One and 10 bp deletions in RS20015 and *pho*R, respectively, along with a 10 bp insertion in RS08265, were also detected in Danish 1331 compared to *M. bovis* AF2122/97.

### Presence or absence of antigenic epitopes

Here, we examined 486 experimentally verified human T cell epitopes in the *M. tuberculosis* H37Rv. Our findings showed that 172 epitopes (35.4%) were absent in both BCG strains (Table S6). Moreover, the Pasteur 1173P2 has lost four additional epitopes relative to Danish 1331, which is associated with the RD14. Most of the absent epitopes from both strains (133 epitopes, 77.3%) are related to the RD1, which was deleted from all BCG strains between 1908 and 1921 [1]. The 23 deleted epitopes were located in RD2 containing the immunogenic protein Mpt64, which is absent only in late strains (obtained after 1927) [1]. Other missing epitopes belonged to RD7 (*n* = 4), RD3 and RD10 (*n* = 3), RD13 (*n* = 2) and RD4, RD5, RD11, and IS*1081* (*n* = 1). A total of 42 antigens in both strains, four in Pasteur 1173P2 and one in Danish 1331, have at least a genetic variation outside their epitopic regions (Table 2). While the antigenic epitopes appear to be conserved amino acid sequences, we found four amino acid substitutions (three nSNPs and one sSNP) in six epitopes in both strains (Table 3). A transmembrane protein (Protein ID: NP_216249.1) had the highest variant-carrying epitopes. A missense mutation in Rv1733c substitutes an uncharged hydrophilic amino acid with a positively charged one in position 68 of the protein chain.

**Table 2:** *M. tuberculosis* antigens containing variants in non-epitope sequences in two BCG strains

Epitope ID	H37Rv locus tag	Gene name	Product	Genetic variation	Pasteur 1173P2	Danish 1331
2190	Rv0171	*mce*1C	Mce family protein Mce1C	nSNP^a^	+	+
4002	Rv3018c	PPE46	PPE family protein PPE46	nSNP	+	-
4520	Rv2627c	-	Hypothetical protein	nSNP	+	+
9474	Rv2608	PPE42	PPE family protein PPE42	nSNP	+	+
18,059	Rv1291c	-	Hypothetical protein	sSNP^b^	+	+
24,566	Rv1037c	*esx*I	ESAT-6 like protein EsxI	nSNP	+	-
32,710	Rv3467	-	Hypothetical protein	nSNPs	+	+
32,860	Rv0670	*end*	Endonuclease IV	sSNP	+	+
39,011	Rv3804c	*fbp*A	Diacylglycerol acyltransferase/mycolyltransferase Ag85A	sSNP	+	+
45,757	Rv0170	*mce*1B	Mce family protein Mce1B	nSNP	+	+
55,156	Rv1945	-	Hypothetical protein	sSNPs & nSNPs	+	+
55,188	Rv1641	*inf*C	Initiation factor IF-3	nSNP	+	+
55,191	Rv3689	-	Transmembrane protein	nSNP	+	+
55,192	Rv3378c	-	Diterpene synthase	sSNP & nSNP	+	+
55,315	Rv2823c	-	CRISPR-associated protein Cas10/Csm1	Insertion	+	+
55,334	Rv2476c	*gdh*	NAD-dependent glutamate dehydrogenase	nSNP & Deletion	+	+
57,680	Rv0174	*mce1*F	Mce family protein Mce1F	sSNPs	+	+
60,095	Rv3714c	-	Hypothetical protein	sSNP	+	+
64,663	Rv0169	*mce*1A	Mce family protein Mce1A	nSNPs	+	+
92,817	Rv1886c	*fbp*B	Diacylglycerol acyltransferase/mycolyltransferase Ag85B	nSNP	+	+
93,270	Rv3839	-	Hypothetical protein	nSNP	+	+
99,857	Rv2770c	PPE44	PPE family protein PPE44	sSNP & nSNP	+	+
99,866	Rv2770c	PPE44	PPE family protein PPE44	sSNP & nSNP	+	-
118,590	Rv2600	-	Integral membrane protein	Deletion	+	+
120,392	Rv1866	-	Hypothetical protein	sSNP & nSNP	+	+
120,408	Rv3883c	*myc*P1	Membrane-anchored mycosin	sSNP & nSNP	+	+
120,481	Rv0934	*pst*S1	Phosphate ABC transporter substrate-binding lipoprotein PstS	nSNP	+	+
120,887	Rv3736	-	AraC/XylS family transcriptional regulator	sSNP & nSNP	+	+
121,059	Rv0755c	PPE12	PPE family protein PPE12	sSNP & nSNP	+	+
125,165	Rv1361c	PPE19	PPE family protein PPE19	sSNPs & nSNP	+	+
126,028	Rv3296	*lhr*	ATP-dependent helicase	nSNPs	+	+
126,912	Rv0024	-	NLP/P60 family protein	Deletion	+	+
140,543	Rv2006	*ots*B1	Trehalose-6-phosphate phosphatase OtsB	sSNPs & nSNP	+	+
140,561	Rv1997	*ctp*F	Cation transporter ATPase F	sSNP & nSNP	+	+
140,576	Rv2780	*ald*	L-alanine dehydrogenase	Deletion	+	+
140,597	Rv3499c	*mce*4A	Mce family protein Mce4A	sSNP	+	-
140,615	Rv2531c	*adi*	Amino acid decarboxylase	sSNP	+	+
140,617	Rv2813	-	Hypothetical protein	nSNP	+	+
144,870	Rv1769	-	Hypothetical protein	sSNP	-	+
161,402	Rv0787	-	Hypothetical protein	nSNP	+	+
168,735	Rv1789	PPE26	PPE family protein PPE26	sSNP	+	+
196,087	Rv3343c	PPE54	PPE family protein PPE54	sSNPs & nSNPs	+	+
229,047	Rv1009	*rpf*B	Resuscitation-promoting factor RpfB	nSNPs	+	+
738,104	Rv3616c	*esp*A	ESX-1 secretion-associated protein EspA	nSNPs	+	+
851,000	Rv1626	-	Two-component system transcriptional regulator	sSNP	+	+
857,468	Rv3792	*aft*A	Arabinofuranosyltransferase	sSNP	+	+
1,081,150	Rv0442c	PPE10	PPE family protein PPE10	nSNPs	+	+

^a^Non-synonymous SNP

^b^Synonymous SNP

**Table 3:** *M. tuberculosis* antigens containing variants in epitope sequences in two BCG strains

Epitope ID	H37Rv locus tag	Gene name	Product	Epitope sequence	Genetic variation	Amino acid substitution	Pasteur 1173P2	Danish 1331
20,707	Rv3497c	*mce*4C	Mce family protein Mce4C	GK**T**YDAYFTDAGGITPG	nSNP^a^	Thr > Pro	+	+
106,585	Rv2628	-	Hypothetical protein	KVQSATIYQVTDR**S**H	nSNP	Ser > Leu	+	+
120,511	Rv0956	*pur*N	Phosphoribosylglycinamide formyltransferase PurN	ETLHERIKVTERRLLVAAVAALAT**H**	sSNP^b^	His > His	+	+
153,959	Rv1733c	-	Transmembrane protein	AAAGTAV**Q**DSRSHVYAHQAQ	nSNP	Gln > His	+	+
155,973	Rv1733c	-	Transmembrane protein	TVSLLTIPFAAAAGTAV**Q**DS	nSNP	Gln > His	+	+
434,619	Rv1733c	-	Transmembrane protein	IPFAAAAGTAV**Q**DSRSHVYAHQAQTRHP	nSNP	Gln > His	+	+

^a^Non-synonymous SNP

^b^Synonymous SNP

Fifty-four antigenic epitopes from *M. bovis* AF2122/97 were also examined in both strains (Table S7). Our findings revealed that nine epitopes (16.6%) were deleted from both strains related to RD1. Of the 45 antigens present, only one non-polar to polar amino acid substitution was found to be outside the epitope sequence of Eis N-acetyltransferase.

## Discussion

There is sufficient evidence that BCG strains have undergone variations in their genomes since they were derived from the parental BCG. Genetic differences among the BCG strains have been considered as one of the factors associated with the vaccine variable efficacy [22], but screening of these differences is a prerequisite for demonstrating the variation in the vaccine efficacy. In this study, we attempted to extract the genetic differences between BCG Pasteur 1173P2 and BCG Danish 1331 with *M. tuberculosis* H37Rv and *M. bovis* AF2122/97 based on whole genome sequencing (WGS) data. According to the latest update of the BCG World Atlas in 2020 [21], Danish 1331 is the most common BCG vaccine strain used in 27 countries (16.6%) worldwide. Pasteur 1173P2 is in the second place with a frequency of 15 countries (9.2%). However, there is no evidence as to which vaccine is superior to the others, and therefore different BCG strains are used in immunization programs around the world. In 2007, WGS of the Pasteur 1173P2 was performed using the Sanger sequencing (ABI3700) of the pUC19, pMAQ1b, and M13 libraries, and a whole-genome shotgun library prepared in the pCDNA2.1 [14]. The WGS of the Danish 1331 was also recently performed by combining a second (Illumina MiSeq) and third (PacBio) generation technologies [23]. Consistent with Brosch’s study [14], we reported 2211 and 694 SNPs in BCG Pasteur 1173P2 compared to the *M. tuberculosis* H37Rv and *M. bovis* AF2122/97, respectively. However, another study using NimbleGen detected 1010 SNPs between BCG Pasteur and *M. tuberculosis* H37Rv; Of them, 945 were correctly identified compared to the whole genome sequence [24]. In addition, the study showed that the NimbleGen method has a limited ability to identify SNPs. Unlike the previous study that reported 42 SNPs in Danish 1331 compared to the Pasteur 1173P2 [23], we found 30 SNPs. This discrepancy is probably due to the inability of short read-based sequencing techniques to identify variants in the PE_PGRS genes. In addition, large indels (i.e., RDs) cannot be accurately examined using short reads alone [23]. The ideal to identify variants and RDs is to use hybrid sequencing platforms that create long read along with short reads, which can minimize variants due to the sequencing errors and provide the correct report for genomic variations and large differences [23]. Due to the presence of inherently repetitive structures in the mycobacterium genome, the use of short reads alone may falsely identify these structures as large indels [23]. Therefore, to prevent incorrect reports, we only examined the RDs and duplications that were previously reported.

Deletion of MB0097c-MB0098c, as a Danish determinant, was identified by the current study as described by Abdallah et al. [25]. Inconsistent, this deletion was not detected by Borgers in the Danish 1331 sequence [23]. We also identified this deletion in the Pasteur 1173P2 with different length, which was not reported by Abdallah et al. [25]. Furthermore, we detected RD Denmark/Glaxo, which truncated PPE33 (Rv1809) and removed Rv1810 (equivalent to MB1840 from *M. bovis*) for Danish 1331, as described by Abdallah et al. [25]. Consistent with the previous study [23], we identified a DU1-like region with a length less than Pasteur 1173P2 DU1. Borgers et al. reported that only Danish 1331 deposited as the WHO reference at the National Institute for Biological Standards and Control (NIBSC) contains this duplication [23]. DU1 has also been reported with different lengths in BCG China and Birkhaug [24]. Moreover, a triploid 7.2 kb DU1-like sequence that covered six genes and crossed the *ori*C region has been identified in a clinical BCG strain (BCG 3281) [26]. The presence of *ori*C in these duplications may indicate that this region is prone to duplicate. The effect of *dna*A-*dna*N (located in DU1) copies on the biology of BCG strains is not well elucidated [13].

Bedwell et al. identified two separate genetic populations in a commercial preparation of the BCG Copenhagen vaccine (a.k.a. BCG Danish 1331) which differed in the copy number of 77 bp repeat in the *senX3-regX3* region (2 and 3 repeats) [27]. As reported previously by Borgers’s study [23], we also identified only three 77 bp repeats for BCG Danish 1331. In contrast, Magdalena et al. reported two repeats for a BCG Danish vaccine strain provided by M. Lagranderie (Institut Pasteur, Paris, France) [28]. This finding may indicate that the different strains of BCG Danish are in circulation. As the previous studies [27, 28], two repeats were reported at *senX3-regX3* region for Pasteur 1173P2 by the present study.

We described a range of variants in BCG Pasteur 1173P2 and BCG Danish 1331 in this study. All detected variants (SNPs and indels below 100 bp) were reported in BCG strains with the complete genome in the NCBI (variable from one strain to all). Examination of strain-specific variants in other strain (Pasteur 1173P2 and Danish 1331 compared to each other) using contigs alignment with the reference genome showed that: 1) the identified variant in one strain is adjacent to the contigs border in the other strain and is not detectable. 2) The variant in one strain is associated with a deleted region in another. 3) The variant in one strain is intact in the other one.

We found the single nucleotide substitutions C592620A and G2074890T (position in *M. tuberculosis* H37Rv) in Danish 1331. While the former leads to an amber termination codon (TAG) at position 323 of the amino acid sequence and premature termination, the latter does not alter the structure of the protein produced. SNP in *gal*E2 (C592620A), which is involved in the galactose metabolism (http://genolist.pasteur.fr/TubercuList/), has not been reported in other vaccine strains. In addition to Danish, which shows a 10 bp deletion in *pho*R, other studies have reported that the BCG Glaxo, Sweden, Birkhaug, and Frappier are defective in *pho*R by frameshift mutations with different base pairs [24]. While several studies have reported that *pho*P plays an important role in *M. tuberculosis* virulence, the role of *pho*R in virulence is less understood [29]. A previous study reported mutations in nine genes required for mycobacterial in vivo survival in 13 BCG strains [30], all identified in this study as well. Except for the identified common SNPs between the 13 BCG strains, we found that the *kdp*D, *lys*X, *pks*12, and *esp*A genes in Pasteur 1173P2 and Danish 1331 carry SNPs that are not present in the other strains. Considering the role of these genes, Pasteur 1173P2 and Danish 1331 may be less resistant to environmental conditions [30], which requires further investigation.

In screening T cell epitopes, we found that several epitopes were lost in Pasteur 1173P2 and Danish 1331 compared to *M. tuberculosis* H37Rv. These findings differ from the results of Zhang et al., which reported 295 epitopes in 13 BCG strains, 117 lost epitopes through deletion of RD1, and 28 of RD2 [31]. A study of 491 experimentally confirmed human T cell epitopes in 21 *Mycobacterium tuberculosis* Complex (MTBC) strains reported that these epitopes are highly conserved compared to the remaining MTBC genomes [32]. They found that this observation contradicts studies in pathogenic viruses, bacteria, and protozoa that showed the genes encoding antigens are highly variable to escape host immunity [33–35]. Although we showed that most of the present epitopes in two strains were as highly conserved as the *M. tuberculosis* strains, we identified amino acid substitutions in six T cell epitopes associated with four antigens compared to the *M. tuberculosis* H37Rv. Replacements in 15 epitopes of nine antigens have been reported by Copin et al., four of which are shared with the present study, including PstS1, Fibronectin-binding protein B/antigen 85B, ESX-1 secretion-associated protein EspA and diacylglycerol acyltransferase/mycolyltransferase Ag85A proteins [36]. All these were reported by Copin et al. as antigens containing variants in their epitopes [36]. Inconsistent, they were identified with variants in their non-epitopic regions in the current study. Moreover, Zhang et al. did not identify mutations in the epitopes of 13 BCG strains [31]. We noted that all absent epitopes in both strains were associated with RDs previously described, and no new missing epitope was identified. We also found that *M. tuberculosis* epitopes present in the BCG strains are highly conserved, probably due to the low rate of genetic variations during in vitro evolution that causes sequence diversity in these regions [36].

## Conclusions

We presented a complete report of the variants (SNPs and short indels) in two of the most widely used BCG vaccines compared to the three reference strains using WGS. These findings may be helpful in a more accurate understanding of phenotypic differences following genetic differences between vaccine strains. In addition, this study may provide evidence for investigating the protective efficacy induced by different BCG strains. This study revealed that the present experimentally confirmed human T cell epitopes are highly conserved in both BCG strains and do not reflect any ongoing evolutionary. However, further studies are required to find the association of genetic variations with vaccine variable efficacy and determine the most effective vaccine strain.

## Methods

### Vaccine strains, DNA isolation and whole genome sequencing

Two strains of the BCG vaccine, Pasteur 1173P2 and Danish 1331, were prepared from Pasteur Institute (Paris, France) and Statens Serum Institute (Copenhagen, Denmark), respectively. The culture of freeze-dried seed lots was carried out on the Sauton broth medium, which is routinely used to produce the vaccine. Genomic DNA preparation was performed according to the protocol described previously [37]. The identity of the vaccine strains was determined using specific primers of DU1 and RD14 for BCG Pasteur 1173P2 [14, 16] and DU2-III for BCG Danish 1331 [14]. Genomic libraries were constructed using a modified Nextera Flex protocol (Hackflex) [38] and sequenced on an Illumina NovaSeq 6000 instrument using an S4 flow cell to generate 150 bp paired-end reads with an average coverage of 133-fold at ithree institute (University of Technology Sydney, Sydney, Australia).

### Genome assembly, alignment, and variant calling

Raw sequence reads were trimmed as a part of the Shovill pipeline (https://github.com/tseemann/shovill) using Trimmomatic (v0.39) [39] to remove adapter sequences and low-quality ends (Phred score < 20) and assessed for quality by FastQC (v0.11.9) [40]. The reads were de novo assembled to construct contigs using Shovill and SPAdes genome assembler (v3.13.1) [41] and put in the GenBank under BioProject No. PRJNA691088 (Pasteur 1173P2 BioSample No.: SAMN17277795; Danish 1331 BioSample No.: SAMN17277808). Filtration was performed [42] to maintain contigs with a size over 1000 bp before evaluating the quality of the assemblies with QUAST (v.5.0.2) [43]. The generated contigs were aligned and rearranged with the *M. tuberculosis* H37Rv (Accession No.: NC_000962.3), *M. bovis* AF2122/97 (Accession No.: NC_002945.4), and *M. tuberculosis* variant bovis BCG str. Pasteur 1173P2 (Accession No.: NC_008769.1) as the reference genomes by Mauve aligner (v20150226) using progressiveMauve algorithm (v20150226) [44]. Annotation was performed using Prokka (v1.12) [45] and Rapid Annotation using Subsystem Technology (RAST) [46, 47]. Raw reads were also mapped to three references mentioned above by Burrows-Wheeler Aligner (BWA) (v0.7.17) [48], followed by BamQC with Qualimap (v2.2.1) [49]. Variant calling was conducted using SAMtools (v0.1.19) [50] to generate VCF output files and Mauve [44]. Raw VCF files were filtered considering the quality score and read depth of more than 30 and 0.75, respectively [25]. The presence or absence of experimentally confirmed human T cell epitopes in the BCG genomes was assessed by retrieving *M. tuberculosis* T cell epitopes from the Immune Epitopes DataBase (IEDB) [51].

## Supplementary Information

**Additional file 1:** **Table S1.** SNPs compared to *M. tuberculosis* H37RV. **Table S2.** Indels compared to *M. tuberculosis* H37Rv. **Table S3.** SNPs compared to *M. tuberculosis* variant bovis AF212297. **Table S4.** Indels compared to *M. tuberculosis* variant bovis AF212297. **Table S5.** SNPs & Indels compared to *M. tuberculosis* variant bovis BCG str. Pasteur 1173P2. **Table S6.** Antigenic epitopes compared to *M. tuberculosis* H37Rv. **Table S7.** Antigenic epitopes compared to *M. tuberculosis* variant bovis AF212297.

## Data Availability

The raw sequence reads can be found in the NCBI SRA database (https://www.ncbi.nlm.nih.gov/search/all/?term=PRJNA691088) under BioProject number: PRJNA691088. The complete genomes of *Mycobacterium tuberculosis* H37Rv, *Mycobacterium bovis* AF2122/97, and *Mycobacterium tuberculosis* variant bovis BCG str. Pasteur 1173P2, which are used as the references in this study, have been deposited in the GenBank with accession numbers: NC_000962.3, NC_002945.4, and NC_008769.1, respectively. All data generated or analyzed during this study are included in this article and its supplementary information files.

## Abbreviations

BCG: Bacillus Calmette–Guérin
TB: Tuberculosis
SNP: Single Nucleotide Polymorphism
InDel: Insertion and Deletion
*M. bovis*: *Mycobacterium bovis*
NTMs: Non-Tuberculosis Mycobacteria
*M. tuberculosis*: *Mycobacterium tuberculosis*
ECBS: Expert Committee on Biological Standardization
CDS: Coding Sequence
RD: Region of Difference
nSNP: Non-synonymous SNP
ORF: Open Reading Frame
sSNP: Synonymous SNP
WGS: Whole Genome Sequencing
NIBSC: National Institute for Biological Standards and Control
MTBC: *Mycobacterium tuberculosis* Complex
RAST: Rapid Annotation using Subsystem Technology
BWA: Burrows-Wheeler Aligner
IEDB: Immune Epitopes DataBase
